# Multispecies Niche Overlap: Moving Beyond Pairwise Metrics to Understand Community‐Wide Similarities in Resource Use

**DOI:** 10.1002/ece3.73886

**Published:** 2026-06-21

**Authors:** Cody M. Kent, Scott Powell, Thomas W. Sherry

**Affiliations:** ^1^ Biology Department Luther College Decorah Iowa USA; ^2^ Department of Biological Sciences George Washington University Washington DC USA; ^3^ Department of Ecology and Evolutionary Biology Tulane University New Orleans Louisiana USA; ^4^ Biological Sciences Department Dartmouth College Hanover New Hampshire USA

**Keywords:** diffuse competition, interspecific competition, niche overlap, niche partitioning, niche space, percent overlap

## Abstract

Measures of niche overlap—particularly in terms of resource use—are important tools in a wide range of ecological and evolutionary studies. A variety of these overlap indices are used, but all are pairwise measures. Yet, most communities are more complex, suggesting the potential value of measures that can be extended to the simultaneous overlap among more than two species. Here we extend percent overlap to any number of potentially overlapping species and then partition the amount of overlap attributable to any specific subset of species. We then apply this partitioned multispecies percent overlap metric to both simulated and empirical communities. Additionally, we provide recommendations on the use and extension of this metric for a variety of research goals across topics in both ecology and evolution, illustrating how this methodology may capture interactions and structures of niche space in ways that traditional, pairwise metrics overlook. Such potential insights include identifying foraging guilds, measuring the degree of diffuse competition, and providing overall niche overlaps within and across phylogenetic clades.

## Graphical abstract

Measures of niche overlap are important tools in various ecological and evolutionary studies. Here we create and apply a multispecies overlap metric to both simulated and empirical communities, illustrating how this methodology may capture interactions and structure of niche space in ways that traditional, pairwise metrics overlook.

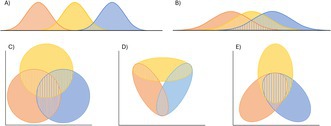

## 1 Introduction

Characterizing and comparing species' niches has played a prominent role in ecology. An important aspect of this work has been quantifying the degree of niche overlap in resource use between pairs of species. Among the many applications of such metrics are describing species similarity (e.g., Morisita 1959; Pianka 1973) and testing for the potential of ongoing competition (e.g., Dhondt 2012; Prins 2016; Kent et al. 2022). Quantifying niche overlap also has value outside of competition, such as characterizing evolutionary trajectories (e.g., Pastore et al. 2021), quantifying species differences (Geange et al. 2011), or in understanding community structure (e.g., Abrams 1983). A variety of metrics and indices have been proposed to characterize the degree of niche overlap in resource space (e.g., Morisita 1959; Horn 1966; MacArthur and Levins 1967; Schoener 1970; Pianka 1973; Hurlbert 1978). This work continues to the present, particularly in efforts to include an increasing number of potential niche axes (e.g., Geange et al. 2011; Swanson et al. 2015). However, although these advances acknowledge the complexity of niche space, metrics still involve species pairs, potentially missing the overall complexity of the communities.

Ecology as a field continues to struggle with the complexity of natural communities, with past work on competition demonstrating that it is difficult to extrapolate from interacting dyads to the more complex communities that are typical in the real world (Moen 1989; Levine et al. 2017; terHorst et al. 2018). However, we recognize that these complexities can be important, such as higher‐order interactions (Levine et al. 2017; Mayfield and Stouffer 2017), or other forms that may be non‐additive (Moen 1989). Similarly, as species pairs typically do not exist in a vacuum, studies based on pairwise niche overlaps may miss potentially important drivers and consequences of community structure in terms of resource use.

**FIGURE 1:**
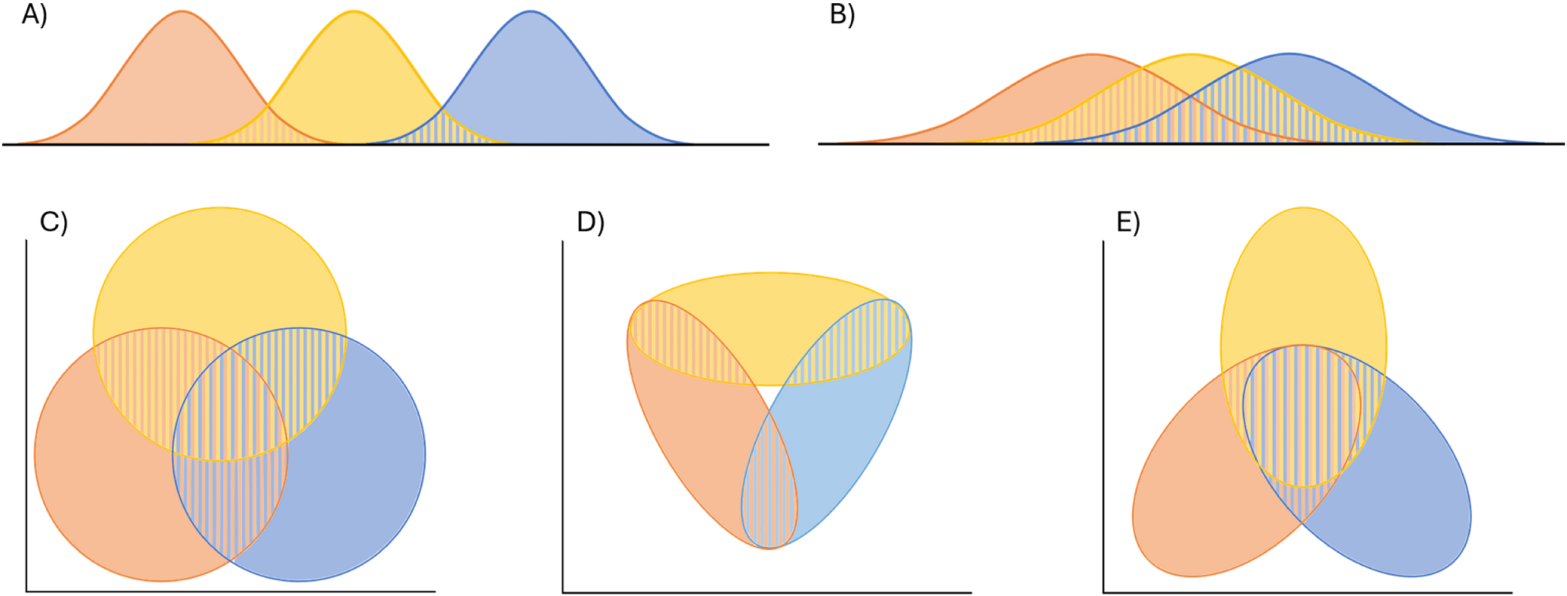
Several potential community structures for three species in niche space. (A) The curves here represent the frequency of use of resources along this axis by three species. Some communities are composed of species that occupy different areas of niche space distributed along a single, linear niche axis. In such communities, most of the overlap may be between adjacent species with less overlap between non‐adjacent species. (B) As niche breadth expands relative to the niche axes, we would expect to see overlap increase among species, including areas of resource space utilized by more than two species. (C) Other communities may similarly have niche overlap concentrated between similar species pairs, but with a more complex underlying resource distribution allowing for more complicated interactions. The ellipses here should be taken to represent the core niche area (such as a 95% confidence interval) of niche space from ordination of various discrete resource states. (D) In some cases, species' niches may be organized such that very little of the niche space is used by all three species despite areas of overlap between all species pairs. (E) Alternatively, in some communities, niche overlaps may be concentrated in areas of niche space where all species overlap heavily, with very little of the available niche space occupied solely by overlapping species pairs.

Moving beyond metrics of niche overlap involving pairwise interactions may provide a fuller picture of species interactions in niche space—particularly in the different resource states they utilize, be that prey items, habitat types, or other potentially limiting resources. For example, many studies of competition examine traits associated with a single, simple continuous niche axis along which species are distributed (Figure 1A), such as seed sizes consumed by Galapagos finches (de León et al. 2014). Here we see some level of overlap between species that are adjacent to each other in niche space, but limited overlap between non‐adjacent species. However, as niche breadth expands relative to the range of the niche axis, we can begin to see areas of niche space used by more than just two species (Figure 1B). Alternatively, some communities may be better characterized by species that overlap with others across more than one axis or on a variety of discrete resources. The potential interactions are perhaps more complex here than in the first example but share similarities. Communities may be organized such that most of the interactions in niche space are between adjacent pairs, but with few resources that are consumed by all members of the assemblage (Figure 1C,D). Alternatively, niche overlaps among species may be high in areas where all species overlap, but with limited pairwise interactions outside of these, as would be the case if all species overlapped heavily on some set of key resources while occupying distinct areas of specialization (Figure 1E).

Here we develop a multispecies overlap metric to better describe and disentangle various community structures, such as those discussed above, in terms of resource use. To accomplish this, we have extended a dyadic metric of percent overlap to any number of species, allowing quantification of the degree of overlap between more than two species simultaneously. We believe this can achieve a better characterization of niche space and overlap in complex communities, which we explore with both artificial and empirical datasets. Due to the wide range of niche overlap applications—from interspecific competition to evolutionary convergence—this multispecies approach may facilitate understanding of other ecological and evolutionary processes in communities, such as adaptive radiation, niche redundancy, and species invasions.

## 2 Methods

### 2.1 Multispecies Niche Overlap

Many metrics of pairwise niche overlap in resource use are available, of which percent overlap (Schoener 1970) is one of the simplest. We developed our partitioned multispecies percent overlaps with this metric due to its straightforward calculation, simplicity of extension to multispecies interactions, and intuitive interpretation, although other metrics may be equally or more valid depending on the system or question. Additionally, as it is less sensitive to the subdividing of resource states (Abrams 1980), percent overlap is useful for comparisons made here with both continuous and discrete resource axes.

Using this metric, percent overlap between two species ($P_{\mathrm{jk}}$) is defined thus:

(1) $$P_{\mathrm{jk}}=1-\frac{1}{2}\left(\sum_{i=1}^{n}\left|p_{\mathrm{ij}}-p_{\mathrm{ik}}\right|\right)$$

where $p_{\mathrm{ij}}$ is the proportional use of resource *i* used by species *j*, $p_{\mathrm{ik}}$ is the use of resource *i* used by species *k*, and *n* is the total number of resource states. When $p_{\mathrm{ij}}$ and $p_{\mathrm{ik}}$ are proportions of the total number of resource states used by each species respectively, this simplifies to

(2) $$P_{\mathrm{jk}}=\sum_{i=1}^{n}\min\left(p_{\mathrm{ij}},p_{\mathrm{ik}}\right)$$

In the case where niche space is measured along a single, continuous axis, where the distribution of resource use for species *j* and *k* can be described by the functions *f*_*j*_ *(i)* and *f*_*k*_ *(i)*, respectively, this is equivalent to the integral under both curves, or $P_{\mathrm{jk}}=\int \min\left(f_{j}\left(i\right),f_{k}\left(i\right)\right)\mathrm{di}$. Usefully for our purposes here, this means that the total niche breadth of each species is scaled to 1, such that 1−$P_{\mathrm{jk}}$ would provide the niche space only occupied by one of the two species.

To extend this metric beyond pairwise interactions, let *A* be a set of *m* species. For a given resource state *i*, let *A*_*i*_ be the set containing the proportional utilization of that resource state ($p_{\mathrm{ij}}$) by all *m* species in set *A*.

(3) $$A_{i}=\left\{p_{i1},\ldots ,p_{\mathrm{im}}\right\}$$

Then, Equation (2) can be extended to *m* species, such that *P*_*A*_ gives the percent overlap among all species included in set *A*.

(4) $$P_{A}=\sum_{i=1}^{n}\min\left(A_{i}\right)$$

However, in characterizing the structure of niche overlap within a community it is likely more useful to describe the percentage of niche space that is occupied and overlapped by some subset of species but not by others, requiring a partitioning of this metric. We can then find the percent overlap between any *m* species that is unique to those *m* species by subtracting out the overlap of all coarser sets of species.

Here we let *B* be a set containing all possible subsets of species, where *A* represents a specific set of species. Finally, let *O*_*A*_ represent the percent overlap of all the species included in set *A*, removing overlap already accounted for in all proper supersets of *A* included in set *B*. We can then order subsets by decreasing rank, defining *O*_*A*_ recursively from the full set downward. In this way, *O*_*A*_ will represent the percent of niche space that is overlapped only by the species included in set *A*. This can be defined as,

(5) $$O_{A}=P_{A}-\sum O_{B|A\subset B}$$

where $O_{A}$ is the overlap between any set of species in the community, and $O_{B|A\subset B}$ is the partitioned overlap for all sets *B* of which *A* is a proper subset. If set *A* is a singleton, $O_{A}$ for species *j* will simplify to the percent of niche space occupied only by itself, as,

(6) $$O_{A}=O_{j}=1-\sum O_{B|j\subset B}$$

such that the percentage of niche space occupied only by itself is the sum of the proportional use of all resource states, which equals 1, minus the sum of all proper subsets that include species *j*. This method also means that the sum of all partitioned overlaps in set *B* will be the number of species (*m*), which may have useful properties in comparisons between communities.

**FIGURE 2:**
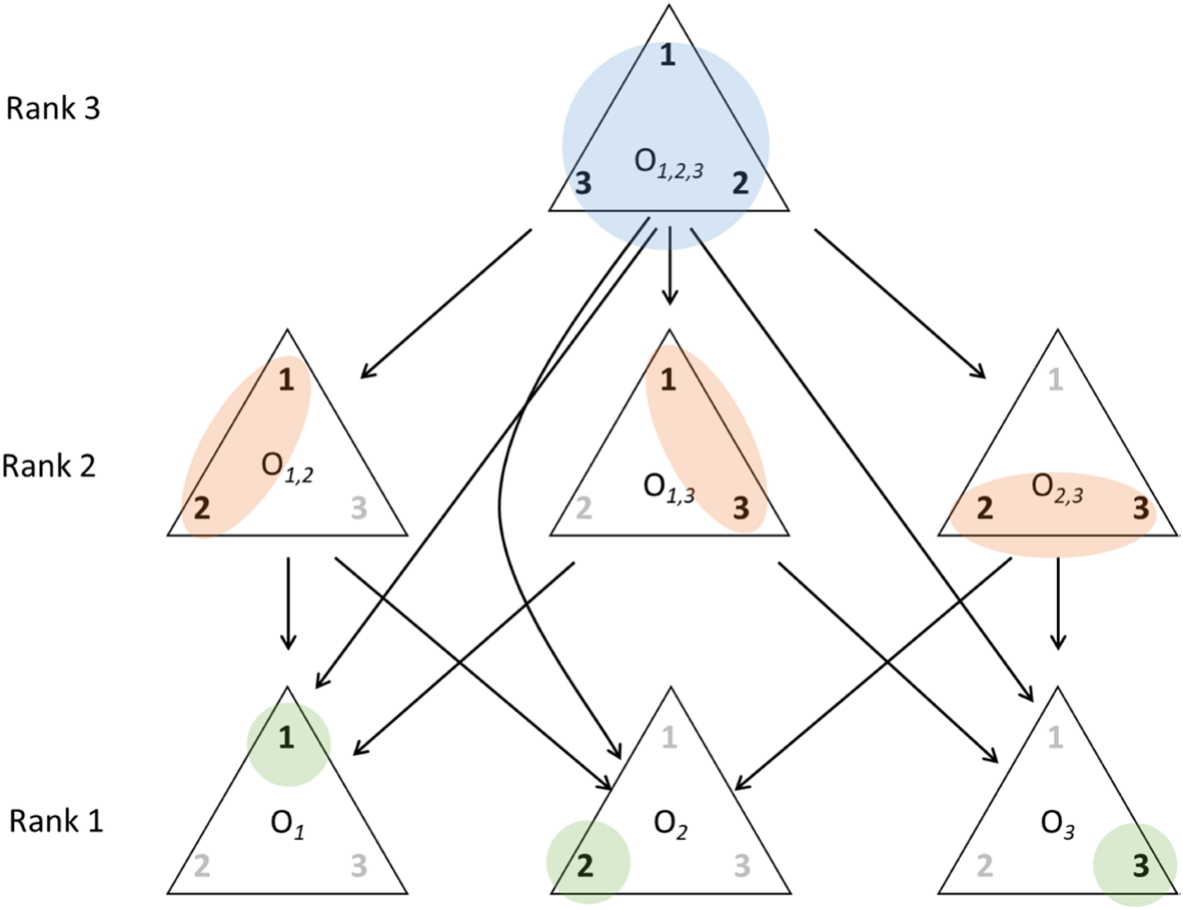
The possible subsets of a community of three species. Decomposition of partitioned overlaps begins at the highest rank (rank 3 here), then recursively subtracts higher‐rank subsets (shown by black arrows) to provide unique overlap contributions for each subset. Values calculated for the first rank (the singleton sets) are the remaining percentage of niche space occupied by each single species after removing overlaps with other species.

Based on Equation (5), we can then solve for all subsets recursively by beginning with the complete set of all species (Figure 2). For example, for a community of four species, we can calculate the overlap among all species by simply using Equation (4),

$$O_{1,2,3,4}=P_{1,2,3,4}$$

Then for all sets of rank *m‐1*, solve based on Equation (5). In this case, for 3 species, this would be,

$$O_{1,2,3}=P_{1,2,3}-O_{1,2,3,4}$$

$$O_{1,2,4}=P_{1,2,4}-O_{1,2,3,4}$$

$$O_{1,3,4}=P_{1,3,4}-O_{1,2,3,4}$$

$$O_{2,3,4}=P_{2,3,4}-O_{1,2,3,4}$$

Which can then be used to calculate all sets of rank *m‐2*, in this case all pairwise overlaps.

$$O_{1,2}=P_{1,2}-\left(O_{1,2,3,4}+O_{1,2,3}+O_{1,2,4}\right)$$

$$O_{1,3}=P_{1,3}-\left(O_{1,2,3,4}+O_{1,2,3}+O_{1,3,4}\right)$$

$$O_{1,4}=P_{1,4}-\left(O_{1,2,3,4}+O_{1,3,4}+O_{1,2,4}\right)$$

$$O_{2,3}=P_{2,3}-\left(O_{1,2,3,4}+O_{1,2,3}+O_{2,3,4}\right)$$

$$O_{2,4}=P_{2,4}-\left(O_{1,2,3,4}+O_{1,2,4}+O_{2,3,4}\right)$$

$$O_{3,4}=P_{3,4}-\left(O_{1,2,3,4}+O_{1,3,4}+O_{2,3,4}\right)$$

Finally, for each set of only one species—the singletons—we apply Equation (6) to represent the percentage of niche space for each species that is not overlapping any other species.

$$O_{1}=1-\left(O_{1,2,3,4}+O_{1,2,3}+O_{1,2,4}+O_{1,3,4}+O_{1,2}+O_{1,3}+O_{1,4}\right)$$

$$O_{2}=1-\left(O_{1,2,3,4}+O_{1,2,3}+O_{1,2,4}+O_{2,3,4}+O_{1,2}+O_{2,3}+O_{2,4}\right)$$

$$O_{3}=1-\left(O_{1,2,3,4}+O_{1,2,3}+O_{2,3,4}+O_{1,3,4}+O_{1,3}+O_{2,3}+O_{3,4}\right)$$

$$O_{4}=1-\left(O_{1,2,3,4}+O_{1,2,4}+O_{1,3,4}+O_{2,3,4}+O_{1,4}+O_{2,4}+O_{3,4}\right)$$

Like the dyadic metric of percent overlap this new metric is based on, this partitioned multispecies percent overlap metric can be extended or modified in various ways. With data availability, percent‐use values could be transformed from raw frequencies to a better measure of their value—such as percent of calories gained from each resource state or percent use relative to their availability. Additionally, although generally designed for percent use of discrete resource states (e.g., prey items or habitats), it can readily be extended to continuous resource states by partitioning the axis into discrete categories. Although not typical in most empirical scenarios, in cases where this continuous resource axis can be described as a function, the same process outlined here can be followed, but substituting the discrete overlap metric (Equation 4) with the continuous form $P_{F}=\int \min\left(F_{i}\right)\mathrm{di}$ where *F*_*i*_ is a set of functions describing the resource states *i* use by each species.

### 2.2 Simulated Communities

To investigate how these metrics perform, we generated three sets of artificial communities with five species each and with different degrees and natures of niche overlap. In the first of these, here called “continuous communities,” species occupy areas of niche space along a single, continuous axis. In this scenario, species' niche use was modeled as truncated normal distributions, bound between 1 and 100, accomplished by setting the probability density function to 0 outside of this bound. The means for each of the five species were fixed for each, ranging from 30 to 70, increasing by 10 for each. The standard deviation, as a metric of niche breadth, was then allowed to vary from 1 to 100.

In the second set of artificial communities, here called “diffuse communities,” each species specializes on a unique discrete resource state, but also overlaps equally with all other species to some extent on their specialized resource states. The degree of niche specificity was allowed to vary based on a ratio of specialization, ranging from 1:1:1:1:1 to 20:1:1:1:1, with the former representing an equal consumption of all resource states by all species and the latter representing a species that consumes 20 of its preferred resource state for every one of the others.

The third set of artificial communities, here called “targeted to diffuse communities”, examines a continuum of diffuse competition where species may overlap more strongly with some species than others. This was done similarly to “diffuse communities”, except that each species shared higher overlap with a unique pair of species and lower overlap with the other two. Here the specialization ratio between a given species and the pair of species it did not have elevated overlap with was set to 10:1. For the remaining two species it was allowed to vary from a ratio of 10:10 to 10:1, giving the total ratio of the species ranging from 10:1:1:1:1, with very even levels of overlap between all species to 10:10:10:1:1, with very uneven levels of overlap between all species. Defined thus, this set of simulations varies from low‐level highly diffuse interactions that are equal among all species pairs to species engaged in strong, pairwise interactions.

### 2.3 Empirical Communities

We also included an analysis of niche overlap in three different communities based on real data to highlight different structures that may exist in nature. Details of the methods used to collect the data for each of these communities are available within their respective citations. Analyses of these three datasets here are primarily for demonstration purposes, and a fuller understanding of the underlying ecology than is provided here would be necessary for proper inference.

The “Cephalotes” dataset comes from published nesting ecology data for coexisting species of turtle ant (genus *Cephalotes*) within an arboreal ant community in cerrado habitat of Brazil (Powell 2008, 2016). Briefly, turtle ants compete for pre‐existing nesting cavities and partition niche space along an axis of entrance hole area (Powell 2008, 2009, 2016). Here we focus on the entrance hole area for the five most common species in the community, which we log‐transformed to normalize the data and better distribute them along our axis for demonstration purposes. This represents partitioning of resources primarily along a single, continuous axis. As the metric requires resource categories, we binned log‐transformed nest entrance diameters into 40 equally spaced groups. This number was selected as it leads to a reasonable number of samples in each resource state while breaking down the data finely enough to illustrate species differences. Fortunately, the percent overlap metric this method is based on is known to be less sensitive to the subdividing of resource states than most other metrics (Abrams 1980).

The “Batoid” dataset comes from Lemos et al. (2024) and analyzes stomach contents of four species of batoid fishes (dorsoventrally flattened elasmobranchs). The dietary data here represent 47 discrete prey categories that are mostly at the family level. These species show fairly low dietary overlaps in general based on a pairwise overlap analysis, with each species showing clear areas of independent specialization with limited overlap with others. This dataset thus represents species that partition resources through unique areas of niche space with limited overlap among all species.

Finally, the “Parulid” dataset comes from Kent and Sherry (2020) and examines dietary data for five species of coexisting arboreal New World wood warblers (family Parulidae) wintering (non‐breeding) in Jamaica. The data consist of 24 categories of invertebrates consumed by these species, with all warbler species showing limited categories of specialization and with large consumption of ants and beetles by all species. This dataset thus represents a diffusely competitive system in which species have discrete specializations alongside common resources consumed at high rates by all members.

## 3 Results

### 3.1 Simulated Communities

**FIGURE 3:**
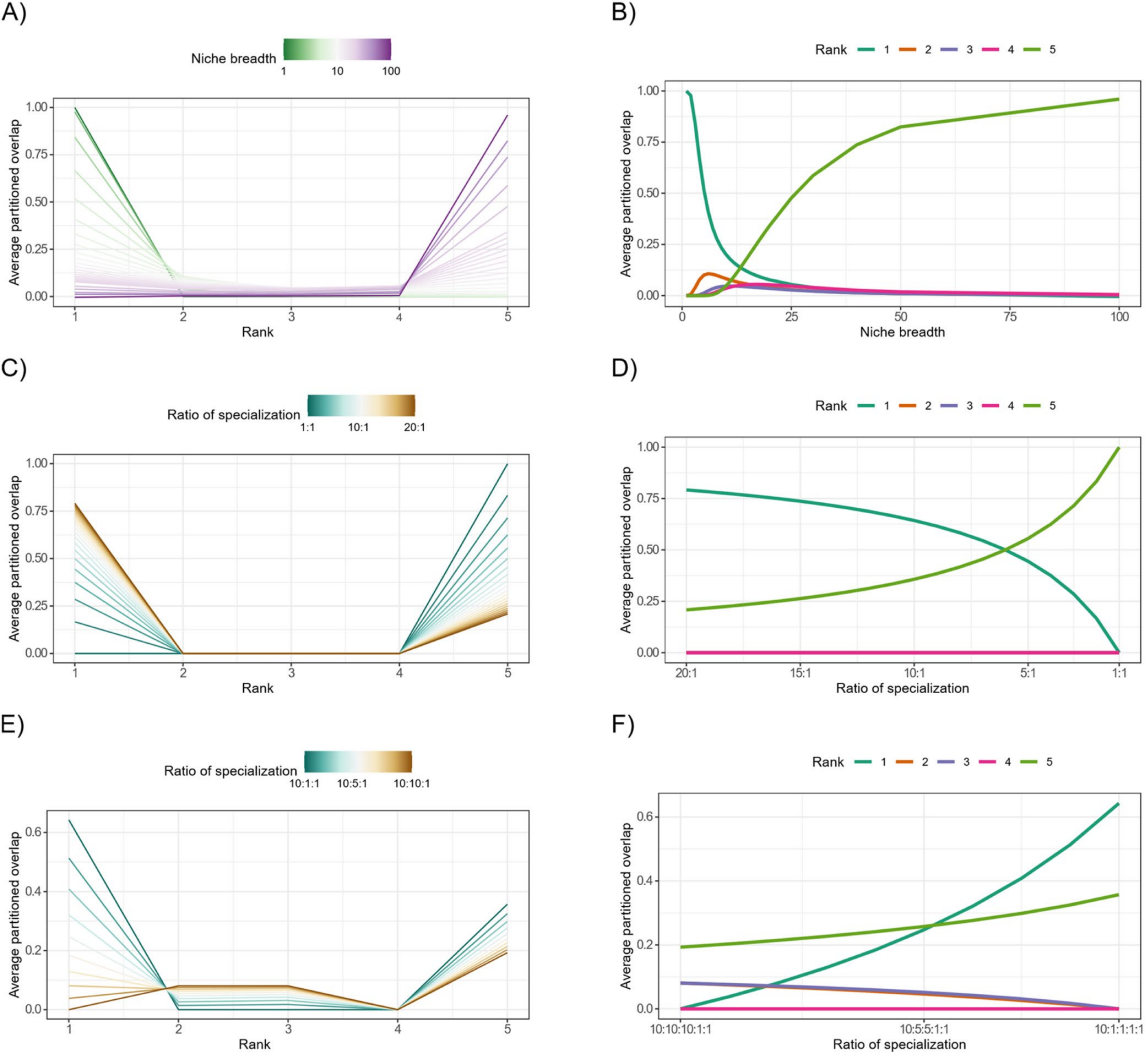
Selected results from simulated communities. (A, B) shows results for “continuous communities,” (C, D) for “diffuse communities,” and (E, F) for “targeted to diffuse communities.” The left‐hand column (A, C, E) shows the average percentage of partitioned overlap (*O*_*A*_) in each of the five ranks, with 1 being non‐overlapping parts of each species' niche space, 2 pair‐wise overlaps, and 5 representing the overlap between all five species in the community. The right‐hand column (B, D, F) shows the change in how much of the total niche overlap is in each rank as niche breadth expands. Note that in C, the first, second, and third ranks all fall on the same line.

In all simulations, as niche breadth expanded, a greater percentage of the overlap was concentrated in coarser subsets of species; however, the nature of these shifts differed among the simulations (Figure 3). For the continuous communities, as niche breadths increased, we first see elevated levels of overlap among species pairs (rank 2, Figure 3A,B), as these are species that occupy niches adjacent to each other along this single, continuous, linear niche axis. As we increased the degree of niche breadth further, we briefly saw bumps of elevated niche overlap pass through the lower and intermediate ranks (ranks 3–4), as overlap became heavily concentrated among all species (rank 5). In the “diffuse” communities, we simply saw a shift between the singleton sets (rank 1, no overlap/unique areas of niche space) to the highest rank involving all species (Figure 3C,D). This makes sense, as we did not have any specifically pairwise interactions here, instead having the area of overlaps being consistent across all species. We saw differing patterns in the “targeted to diffuse communities,” where there were pairs of species that overlap more heavily than the rest of the community—although the degree of these targeted interactions varied (Figure 3E,F). Here we find that as competition became more diffuse, a higher concentration of overlap occurred among all species, while for those that were more targeted among sets of two overlapping species, more overlap occurred at lower ranks.

### 3.2 Empirical Communities

**FIGURE 4:**
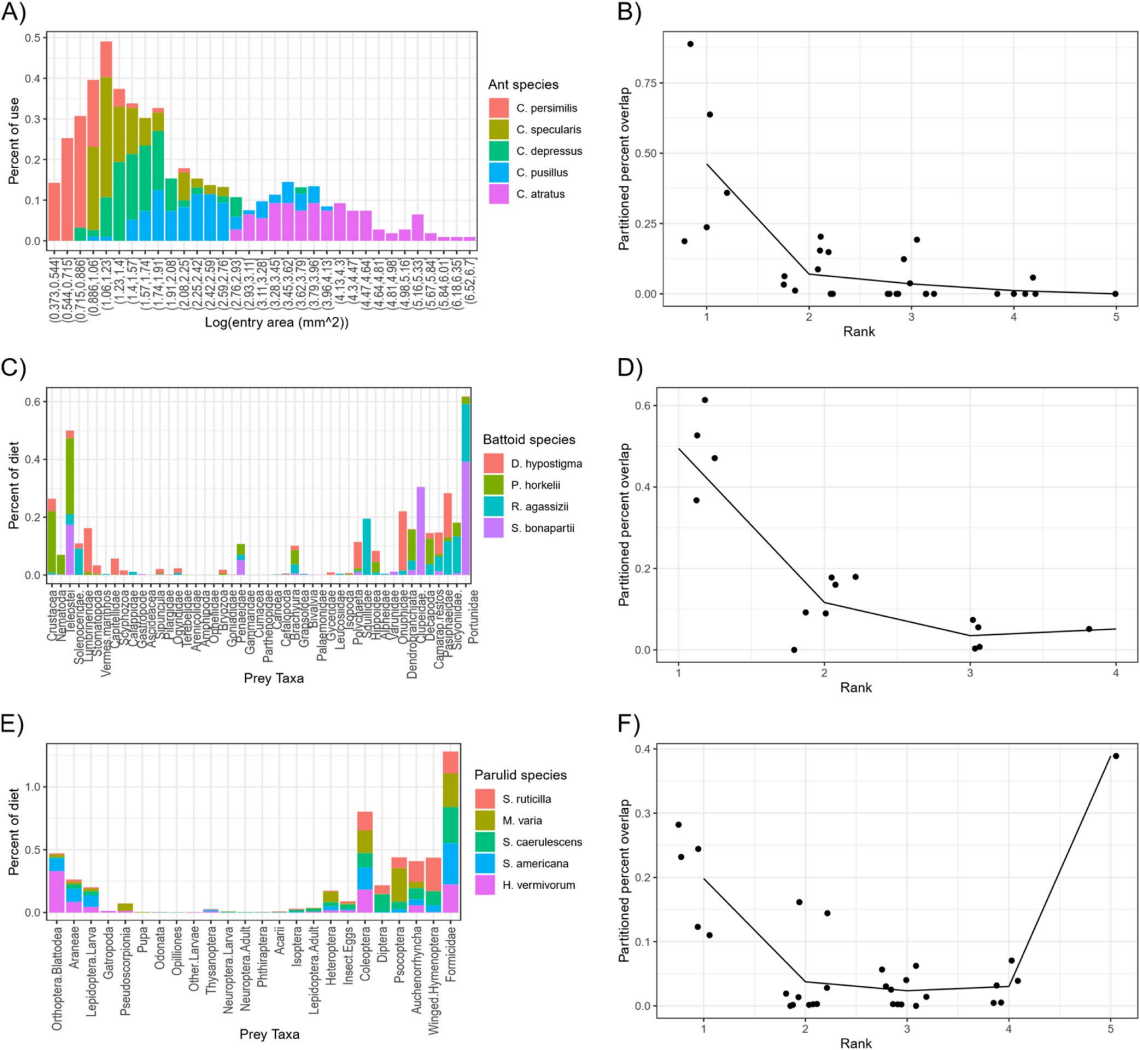
Partitioned multispecies percent overlap in empirical communities. *Cephalotes* (A, B), batoids (C, D), and parulids (E, F). Bar plots (A, C, E) show the percentage use of different resource states for each species. Right‐hand plots (B, D, F) show the percentage of partitioned niche overlap (*O*_*A*_) at each rank. Points indicate actual overlaps, which have been jittered horizontally to prevent overplotting. Lines show the average overlap for subsets of different ranks.

The three empirical communities show different patterns of resource overlap across the higher ranks (2–5). Although these overlaps differed in magnitude, we were more interested here in how they differed in overall structure. *Cephalotes* ants, which partition nesting space along a single, continuous axis of entrance hole area (Figure 4A), showed a steady decline in overlaps as rank increased (Figure 4B), with several pairwise overlaps accounting for greater than 15% of niche space and falling to zero overlap in niche space among all five species.

In the batoids, overlap was generally limited, but that which existed was mainly between species pairs (rank 2, Figure 4D). All four species overlapped on only 5% of the total niche space, while on average each species had roughly 50% of its overall niche space that was not overlapping competitors and some pairwise overlaps reaching 14%–16%. This is consistent with most species specializing on a handful of specific prey with some overlap with species occupying related niches (Figure 4C). For instance, *R. agassizii* and *S. bonapartii* both consumed large numbers of Sicyonnidae (prawns), *D. hypostigma* and *R. agassizii* both consumed measurable amounts of Pasiphaeidae (shrimp), and *P. horkelii* and *S. bonapartii* both consumed larger numbers of Teleostei (ray‐finned fishes). However, there were no prey items that were consumed to a large degree by all four species.

Finally, in the parulid warblers, most of the overlap (39% of total niche space) was located in the coarsest set (rank 5, Figure 4F), along with some areas of unique specializations (averaging 20% of each species' total prey resource space). This appears to be driven by all species consuming large numbers of ants (Formicidae) and beetles (Coleoptera), with more limited pairwise overlaps (rank 2, Figure 4E), although there were still some notable pairwise interactions. Specifically, this community contained two pairs with elevated overlaps, with each pair having greater than a 10% overlap. This occurred between *S. ruticilla* and *S. caerulecsens*, with both consuming a moderate number of flies (Diptera) and winged hymenopterans; as well as between *S. americana* and *H. vermivorum* , with both consuming spiders (Aranea) and crickets/cockroaches (Orthoptera/Blattodea).

## 4 Discussion

We have described here a novel metric of multispecies percent overlap that can be applied to any number of species. Although communities are often complex, with many potentially overlapping species occupying different areas of niche space, traditional niche overlap metrics have only examined these in a pairwise fashion. This may overlook other interesting and important patterns that may lead to distinct ecological and evolutionary outcomes. As such, understanding these diverse communities and potential outcomes necessitates a relevant quantitative approach. Below we first describe our general findings for the simulated and empirical datasets examined here. This is followed by recommendations of how to use this metric and how to extend it to multiple niche axes, before returning to the empirical datasets examined here to explore potential eco‐evolutionary consequences and dynamics.

### 4.1 Capturing Niche Overlap Patterns

We applied this multispecies percent overlap metric to three real communities as well as sets of simulated communities to demonstrate different patterns of niche overlap. In both our simulated and empirical communities, we first examined communities where niche space is divided along a single, continuous, linear axis. Although relatively simple, such communities have been common study systems, such as seed size in Darwin's finches (de León et al. 2014) or water depth for barnacles (Connell 1985). Here we examined five coexisting species of *Cephalotes* ants, which subdivide niche space primarily along a continuous axis of nest entrance hole size (Powell 2008, 2016). These ants compete for limited nesting cavities, the entrances of which they defend with head armor that aligns with the typical size and degree of specialization in the holes that each species finds and uses. Such systems often follow some form of ordinal partitioning (e.g., small, medium, and large), in which most of the overlap is between species adjacent to each other in niche space. So, for example, here small species that use small entrance holes may overlap with medium‐sized species that use medium‐sized entrance holes but likely interact much less with larger species that use larger ones. Consistent with this, most of the overlap that existed in this system was pairwise, with no resources that were shared among all species. In such systems, this relationship theoretically breaks down as niche breadth becomes wide enough, as shown by our simulations (Figure 3A). As niche breadth increases dramatically relative to the degree of niche separation and the length of the niche axis, species on opposite ends of these axes may start to overlap with each other, with all species consuming relatively large amounts of resources in the center of the niche axis.

In many communities niche space is not so simply subdivided along a single axis, particularly when individuals consume a variety of discrete resource states that cannot be ordered along any single axis. In our simulations with these communities, when competition is diffuse and niche breadth is large, niche overlap is typically concentrated among all species (e.g., Figure 3C,E). However, in more targeted interactions, where species overlap more with particular community members, we can see more elevated levels of overlap in the higher ranks. Here we demonstrated two distinctive forms that niche overlap may take on these discrete resource states. The first of these examined dietary overlap in batoid fishes, in which most of the niche overlap that did exist was concentrated between species pairs, with very limited overlap involving greater numbers of species (Figure 4D). This was driven by most species overlapping with a coexisting species in some distinct area of niche space, but with very few resources that were consumed by all four species and rarely were any consumed to a meaningful degree by any three batoid species. This distribution of resources led to mostly pairwise interactions and similar overall patterns to what we observed in *Cephalotes* ants.

The structure of niche overlaps in the batoid and parulid datasets differed strikingly. In the parulid warblers, most of the overlap in niche space involved all five species, specifically overlapping on ants (Formicidae) and beetles (Coleoptera). However, each warbler species also simultaneously specialized on some distinctive prey categories. One may expect to see such outcomes regularly in communities in which large numbers of species rely heavily on the same resources that may be either relatively abundant, accessible, or of high value. In such systems, species may occupy distinct areas of niche space outside of these specific resources on which they overlap with all other community members.

### 4.2 General Method of Interpretation

Niche overlap metrics are applied to many different questions across both ecology and evolution. As such, their interpretation and applicability are heavily dependent on research questions and study designs. Here we discuss the use of this metric separately across three broad categories for which they are commonly used: interspecific competition, community structure, and evolutionary patterns.

First, one common use of these metrics is to test for or quantify interspecific competition or the level of niche partitioning. Some such studies seek to test for ongoing interspecific competition (e.g., Dhondt 2012; Prins 2016; Kent et al. 2022). Such studies rely on properly identifying relevant niche axes—typically done by documenting that both (1) a resource is limiting and (2) that lack of a resource has fitness consequences before then documenting (3) overlap in resource use to demonstrate ongoing competition (Dhondt 2012; Prins 2016). Depending on the study design and knowledge of the system, high levels of overlap may then be interpreted to either indicate the potential for ongoing interspecific competition or that a specific niche axis is not important for niche partitioning and coexistence. Historically in such studies it has been regularly asked if there is some minimum level of similarity that is allowable for coexistence in a competitive context. For the metric proposed here, we generally caution against the assumption that any level of overlap is “too much” to allow for coexistence, since this metric alone—like most niche overlap metrics—does not consider whether resources are in fact limiting to the relevant populations. Future empirical work will be needed to understand if there are certain levels or patterns of overlap across multiple species that appear to be uncommon, and thus unlikely in real communities.

In such a competition study, the multispecies overlap metric we propose here would allow a researcher to further quantify the degree to which there is diffuse competition among all individuals competing for the same underlying resource (e.g., Kent et al. 2022) or more targeted competition isolated to species pairs. As an example, here we demonstrate that the nature of the overlap in niche space between the three empirical datasets is markedly different—with far stronger pairwise interactions for the *Cephalotes* and batoid datasets compared to the very diffuse interactions in the parulid warbler datasets (Figure 4B,D,F). Likewise, there has been some argument about whether diffuse competition prevents or promotes species invasions (Stump 2017; Godoy 2019), differences that may in part be linked to how diffuse competition is defined and how the whole community overlaps in niche space.

Secondly, as a measure of niche similarity—this metric has novel application for our understanding of community structure. For instance, it could be used to more clearly demonstrate levels of functional similarity, where high overlaps across all species may show high levels of niche redundancy linked to greater community resilience (Biggs et al. 2020). Moreover, this metric would be highly appropriate for understanding guild and related structures in communities (Simberloff and Dayan 1991). Such patterns—where species are grouped based on utilizing the same subset of resources—would be readily identified by specific subsets of species at higher ranks but below the full set of species that show elevated levels of overlap. That is, they would be using a similar group of available resources unique from other groups of species. For example, this metric allowed us to graphically demonstrate elevated levels of overlap between specific pairs of warblers ( *S. ruticilla* —*S. caerulecsens* and *S. americana* — *H. vermivorum* ) that were unique from the rest of the species (Figure 4F).

Thirdly, this metric may allow for novel insight for studies of evolutionary patterns—which often use niche overlap metrics to quantify similarity between species (e.g., Warren et al. 2008). For instance, in an evolutionary context, high levels of niche overlap among all species within a lineage may indicate a high level of niche conservatism, while high overlaps across lineages may demonstrate niche convergence or environmental forcing. In such a context, one potentially interesting application may be to map the niche overlap of relevant subsets of species to the phylogeny, providing insights into contrasting or convergent patterns of overlap within and among subclades. Such an analysis would allow one to document when groups of related species diverged or converged in niche space from their larger clades and subsets of species.

### 4.3 Selecting and Extending Axes

In all research contexts—as is true with all other niche overlap metrics—it is the responsibility of the researcher to determine appropriate axes. This is particularly true in studies that are trying to test for or quantify ongoing competition—where high overlap alone does not necessarily indicate high levels of competition, as species may differ among unmeasured axes (Murray et al. 2023). With this in mind, ecologists have long recognized the multidimensional nature of niche space and increasingly have sought to extend metrics of niche overlap into multiple niche axes. The method we provide here can generally be applied to multiple niche axes through previously developed methods—though again, the appropriate method is dependent on the research context, precluding us from providing a single solution.

In the simplest case—when researchers are using this metric as a measure of niche similarity—as is often the case in evolutionary contexts or broader studies of community structure—it may be appropriate to simply average the overlap for each axis for each subset of species (Geange et al. 2011), such that the average niche overlap (*NO*) for each subset across all niche axes (*T*) would be:

(7) $${\mathrm{NO}}_{A}=\frac{1}{T}\sum_{t=1}^{T}O_{A,t}$$

However, if attempting to quantify some total level of niche overlap, such as when testing for or quantifying ongoing competition, researchers may instead see these niche axes as multiplicative instead of additive. For instance, if two species overlap heavily on one axis, but do not overlap at all on a second, we may assume there is likely no competition between them—necessitating other methods of extending niche overlap into multiple dimensions. In the simplest scenario in which all niche dimensions are truly independent, these overlaps could be multiplied together for each subset of species to provide a multidimensional niche overlap (e.g., Pianka 1974). However, in many cases these are unlikely to be independent, and may necessitate calculating probabilities that a set of niche axes are used jointly at the same time. For instance, Swanson et al. (2015) characterized niche overlaps based on the joint probability of two species using the same set of resource states. Such a metric can readily be adapted to the probability of more than two species using the same set of resource states and then partitioned using the same method described above. Moreover, when measuring across multiple axes, it is worth noting the scenario of overlap in resources that cannot be separated into relevant subgroups or added up—such as plant systems where competition may be primarily for access to light, nitrogen, and phosphorous. Such axes generally cannot be combined in this or similar frameworks and remain a shortcoming of this class of metrics in general. However, in cases where these can be characterized via functional traits (e.g., Mouillot et al. 2005) and quantified via kernel density functions, a similar metric to what is proposed here could be applied.

### 4.4 Ecological and Evolutionary Perspectives

Using these multispecies niche overlaps may lead to better insights into underlying community structures, with potential for informing our understanding of both ecological and evolutionary processes, particularly in systems structured by interspecific competition (Chase et al. 2005; Rabosky 2013; Gillespie et al. 2020). Though research on the role of competition in shaping coexistence patterns has overwhelmingly focused on species pairs (Moen 1989; Chesson 2000; Levine et al. 2017), we may nevertheless expect communities with different structures to shape evolutionary trajectories in different ways. Such patterns may help explain phenomena such as repeated observations of decoupling of trait evolution within a lineage (Stoks et al. 2005; Pélabon et al. 2013) or why some communities seem more susceptible to or impacted by invasive species (Godoy 2019).

In *Cephalotes* ants, the support from our partitioned multispecies percent overlap metric for mostly pairwise overlap in nesting resources is consistent with the macroevolutionary dynamics of the head armor the ants use to defend their nests. The evolution of head armor, expressed in a distinct soldier caste in most *Cephalotes*, follows a macroevolutionary pattern of divergent, speciational jumps (Powell et al. 2020). This divergent pattern of phenotypic diversification, and by extension the specialized nesting resources they defend, is consistent with intense competition over nest entrances among taxa occupying adjacent ecomorphological space. Additionally, and concordantly, theory has shown that competition over specialized nesting resources can drive disruptive selection, evolutionary branching, and evolution of a specialized defensive soldier, consistent with empirical data in *Cephalotes* (Planque et al. 2016). Under ongoing eco‐evolutionary feedback of this kind between ecological and morphological specialization, we would then expect present day communities to display high pairwise niche overlap, with minimal to no interactions at higher ranks: that is, we expect communities with ordinal partitioning of relatively narrow niches along the continuous, linear axis of nest entrance size. Our new partitioned multispecies overlap metric recovers precisely this pattern (Figure 4B), reciprocally corroborating the community interactions thought to underlie the identified macroevolutionary dynamics in the group, and suggesting that such interactions are ongoing in present‐day communities.

In contrast, a different eco‐evolutionary pattern prevails in systems such as the parulid warblers. This pattern is instead consistent with a phenomenon known as Liam's paradox, first described in the Lake Tanganyika cichlids (Liem 1980), where despite clear differences in evolved morphology or behavior, high levels of resource overlap persist, particularly on a handful of plentiful or high‐value resources (Robinson and Wilson 1998). This paradox has been resolved through the concept of the “imperfect generalist,” in which species typically forage as generalists but with discrete areas of specialization to allow for coexistence (de León et al. 2014). Such a scenario may arise when interspecific competition for food is high, but the relative abundance of different resource states is unpredictable (Sherry and Kent 2022). In this context, past evolutionary pressures have selected for some degree of resource specialization—either to avoid interspecific competition or to allow for greater foraging efficiency in general—but only to the extent that it does not limit an individual's ability to use other resources—leading to the overlap patterns shown here (Figure 4F). Such a strategy is exemplified by the parulid warblers, in which each of the species presented here has unique morphologies for their different foraging behaviors (especially substrates exploited). For instance, *S. ruticilla* forages in the airspace, *H. vermivorum* in suspended dead leaf clusters, *M. varia* clinging onto bark surfaces, and *S. caerulescens* in a variety of microhabitats in the understory (Rosamond et al. 2020). Likewise, these differences in morphology and foraging behavior do lead to some level of dietary specialization (Kent and Sherry 2020). Nevertheless, these warblers still forage as resource generalists overall, overlapping highly on whatever resources may be available at any time and place—providing them with a degree of foraging flexibility (Sherry et al. 2016; Southwell 2018; Kent and Sherry 2020; Kent et al. 2022; Miller et al. 2025).

This parulid warbler pattern contrasts with that found in batoids, in which species appear to occupy distinct, but sometimes overlapping, areas of niche space. Results like this may arise in systems in which species partition niche space along independent, but potentially intersecting, axes. For instance, one could imagine a system in which species A consumes large prey, species B consumes small prey, species C consumes active prey, and species D consumes sessile prey. We may see overlap between species A and C on large, active prey or B and D on small, sessile prey. In fact, we could see elevated pairwise interactions among all possible species pairs, but no individual resources consumed by all four species. Similarly, within the four batoids discussed here, *D. hypostigma* consumed a subset of benthic worms and shrimp, *R. agassizii* primarily fed on benthic crustaceans (both shrimp and crabs), *S. bonapartii* foraged on a variety of benthopelagic crabs and fish, and *P. horkelii* fed primarily on benthic fish and worms (Lemos et al. 2024). Given these patterns, *D. hypostigma* and *R. agassizii* overlapped on a variety of benthic crustaceans, *R. agassizii* and *S. bonapartii* overlapped on benthic crabs, *S. bonapartii* and *P. horkelii* overlapped on fish, and *P. horkelii* and *D. hypostigma* overlapped on a variety of benthic worms. However, there are no resources that all four species consumed to a high degree (Figure 4D). Outcomes like these may be most likely when niches have evolved relatively independently, and/or over long time periods, among predators in response to distinct ecological pressures, leading to independent and discrete areas of niche overlap and perhaps more predictable evolutionary trajectories.

### 4.5 Conclusions

Here we focused on a single metric of niche overlap, percent overlap, because of its simple interpretation that makes it useful for an initial exploration of the topic. We have shown that the multispecies percent overlap metric we derived can capture a variety of expected differences in simulated communities and distinct community structures across diverse organisms. In so doing, it has deepened our understanding of these focal systems and the overall partitioning of resources within them, as well as contributed perspective and corroborating support for an expanded set of ecological and evolutionary questions in these systems. Next steps may be applying this metric to more systems and extending other niche overlap metrics to include more species. Additionally, a better understanding of the eco‐evolutionary consequences of these differences in community structure is necessary. For instance, although models of interspecific competition at the population level focus on pairwise interactions (e.g., Lotka‐Volterra competition models), more work needs to be done on modeling these more complex interactions. One particularly useful line of inquiry may be focusing on agent‐based, or resource‐state based models in which the nature of resource use across the community can be better controlled.

Improving our understanding of the eco‐evolutionary impacts of these different community structures may also play an important role in shaping our understanding of how communities arose and may adjust to a changing world today. As such, application of this metric to topics ranging from phylogenetic divergence to invasion ecology may provide novel insights. Disentangling these and other central questions in community ecology necessitates expanding not just our study systems but also our metrics, beyond simple species dyads to better examine complex communities as a whole.

## Author Contributions

**Cody M. Kent:** conceptualization (lead), data curation (equal), formal analysis (lead), funding acquisition (equal), investigation (lead), methodology (lead), project administration (lead), software (lead), visualization (lead), writing – original draft (lead), writing – review and editing (equal). **Scott Powell:** data curation (equal), funding acquisition (equal), writing – review and editing (equal). **Thomas W. Sherry:** conceptualization (supporting), data curation (equal), writing – review and editing (equal).

## Funding

This work was supported by Division of Environmental Biology, 2312889.

## Conflicts of Interest

The authors declare no conflicts of interest.

## Data Availability

The data and code necessary to reproduce all analyses presented here will be made available on figshare: https://doi.org/10.6084/m9.figshare.29641085.
